# Attempted Breastfeeding Before Hospital Discharge on Both Sides of the US-Mexico Border, 2005: The Brownsville-Matamoros Sister City Project for Women’s Health

**Published:** 2008-09-15

**Authors:** Brian C Castrucci, Leticia E Piña Carrizales, Denise V D'Angelo, Jill A McDonald, Juan Acuña, Hillary Foulkes, Ginger L Gossman, Kathy Clatanoff, Kayan Lewis, Gita Mirchandani, Indu B. Ahluwalia, Tracy Erickson, Brian Smith

**Affiliations:** Office of Title V, Division of Family and Community Health Services, Texas Department of State Health Services; Jurisdiction III, Secretariat of Health, Tamaulipas, Mexico; Division of Reproductive Health, National Center for Chronic Disease Prevention and Health Promotion (NCCDPHP), Centers for Disease Control and Prevention (CDC), Atlanta, Georgia; Division of Reproductive Health, National Center for Chronic Disease Prevention and Health Promotion (NCCDPHP), Centers for Disease Control and Prevention (CDC), Atlanta, Georgia; Division of Reproductive Health, National Center for Chronic Disease Prevention and Health Promotion (NCCDPHP), Centers for Disease Control and Prevention (CDC), Atlanta, Georgia; Office of Title V, Division of Family and Community Health Services, Texas Department of State Health Services, Austin, Texas; Office of Title V, Division of Family and Community Health Services, Texas Department of State Health Services, Austin, Texas; Office of Title V, Division of Family and Community Health Services, Texas Department of State Health Services, Austin, Texas; Office of Title V, Division of Family and Community Health Services, Texas Department of State Health Services, Austin, Texas; Office of Title V, Division of Family and Community Health Services, Texas Department of State Health Services, Austin, Texas; affiliation; Nutrition Services Section, Division of Family and Community Health Services, Texas Department of State Health Services, Austin, Texas; Region 8 Medical Director, Division of Regional and Local Health Services, Texas Department of State Health Services, Harlingen, Texas

## Abstract

**Introduction:**

The US-Mexico border region has a growing population and limited health care infrastructure. Preventive health behaviors such as breastfeeding ease the burden on this region's health care system by reducing morbidity and health care costs. We examined correlates of attempted breastfeeding before hospital discharge on each side of the US-Mexico border and within the border region.

**Methods:**

The cross-sectional study included women who delivered a live infant in Matamoros, Tamaulipas, Mexico (n = 489), and Cameron County, Texas (n = 457), which includes Brownsville, Texas. We interviewed women before hospital discharge from August 21 through November 9, 2005. We used multivariate logistic regression to estimate the odds of attempted breastfeeding before hospital discharge in Cameron County, Texas, the municipality of Matamoros, Mexico, and the 2 communities combined.

**Results:**

Prevalence of attempted breastfeeding before hospital discharge was 81.9% in Matamoros compared with 63.7% in Cameron County. After adjusting for potential confounders, the odds of attempted breastfeeding before hospital discharge were 90% higher in Matamoros than in Cameron County (adjusted odds ratio [AOR], 1.93; 95% confidence interval [CI], 1.31-2.84 for the combined model). In the 2 communities combined, odds of attempted breastfeeding before hospital discharge were higher among women who had a vaginal delivery than among women who had a cesarean delivery (AOR, 1.98; 95% CI, 1.43-2.75) and were lower among women who delivered infants with a low birth weight than among women who delivered infants with a normal birth weight (AOR, 0.26; 95% CI, 0.15-0.44).

**Conclusion:**

The rate of attempted breastfeeding in Matamoros was significantly higher than in Cameron County. Additional breastfeeding support and messages on the US side of the US-Mexico border are needed.

## Introduction

Between 1950 and 2000, the US-Mexico border population increased by approximately 10 million people (1). This growth is expected to continue. Conservative estimates predict a 34% increase in population between 2000 and 2030, and more liberal estimates suggest a 97% increase (2). Population growth on the border has led to quality-of-life improvements such as paved streets and access to education. However, this population growth is also a potential burden on the health care system, which could result in limited health care access and contribute to significant cross-border use of services (3,4). In a region with limited health care infrastructure, increasing the prevalence of preventive health behaviors such as breastfeeding may ease the burden on the health care system by reducing morbidity and health care costs.

Human milk is a more beneficial form of nutrition for infants than formula (5,6). Breastfeeding has proven short-term and long-term maternal and infant health benefits and reduces health care costs (5-10). Infants who are breastfed have reduced incidence and severity of several infectious diseases (5,6). Breastfeeding has been associated with a lower risk of childhood overweight and obesity, diabetes, asthma, and some cancers (5,6). Women who breastfeed experience increased postpartum weight loss, decreased risk of women's cancers, and possibly improved bone health during the postmenopausal period (5,6).

Studies of breastfeeding in the US-Mexico border region have focused on samples from the United States (11-23) and have explored acculturation (11-17) and the effect of nativity, ethnicity, and immigration on breastfeeding rates (18-23). Interest in binational approaches to health promotion is increasing, and information is needed to assess the prevalence and correlates of breastfeeding in the border region. Despite this need, differences in data collection, measurement practices, and confidentiality issues, stemming from legal and cultural restrictions, inhibit the sharing of information across the US-Mexico border (24). We eliminated the challenges of binational data collection by using identical sampling and survey instruments on each side of the US-Mexico border.

The purpose of this study was to determine the rates of attempted breastfeeding before hospital discharge among women who recently gave birth in the US-Mexico border region, using data collected in 1 of 14 pairs of sister cities located on the US-Mexico border: Brownsville, Texas, and Matamoros, Tamaulipas, Mexico (25) (Figure).

**Figure.**
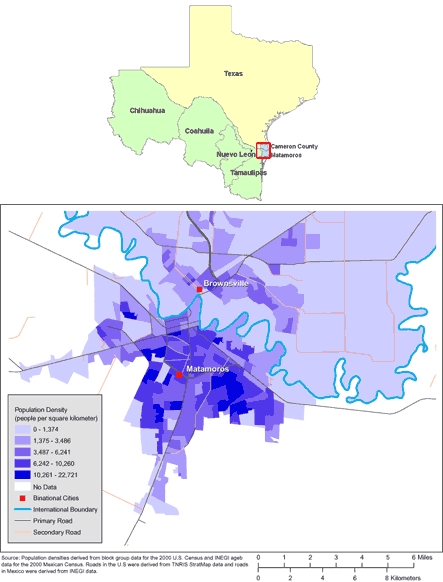
Maps of the US-Mexico Border Region (Top) and of Brownsville, Texas, and Matamoros, Tamaulipas, Mexico (Bottom). (The authors thank Allison Abell Banicki of the Office of Border Health, Texas Department of State Health Services, for creating the map of the Texas-Mexico border states and thank Jean W. Parcher, Sylvia N. Wilson, and the United States Geological Survey [USGS] for providing the map of population density in Brownsville and Matamoros.)

## Methods

### Data collection

We used data that were collected as part of the Brownsville-Matamoros Sister City Project for Women's Health (BMSCP). The BMSCP pilot project was reviewed for human subject concerns by the Centers for Disease Control and Prevention (CDC) and was determined to be "nonresearch" or public health practice. Therefore, institutional review board approval was not required. The study used a stratified, systematic, cluster-sampling probability design to select women who delivered live infants in Matamoros, Mexico, and Cameron County, Texas. Strata were hospitals with 100 deliveries or more per year in either location. We used systematic sampling to select specific days within each stratum, and every woman who gave birth on selected days (within a cluster of days) was included in the sample. Of the 999 women sampled on selected days from August 21 through November 9, 2005 in hospitals with 100 or more deliveries per year, 947 (95%) completed interviews. A more thorough description of the data collection process and other methods used in the BMSCP is available in this issue of *Preventing Chronic Disease* (26).

### Measures

The outcome variable of interest was attempted breastfeeding before hospital discharge. Women were asked, "Have you ever tried to breastfeed your new baby?" One respondent did not provide a response for the outcome variable and was eliminated from the analysis. The final unweighted sample was 946 women, distributed between Matamoros (n = 489) and Cameron County (n = 457). A detailed list of measures used in this study is presented in Table 1.

### Data analysis

We weighted the data to account for probability of selection, population noncoverage, hospital noncoverage, and nonresponse. We used SUDAAN version 9.01 (RTI International, Research Triangle Park, North Carolina) to account for the complex survey design. Data were analyzed by place of residence and for the 2 communities combined. We used the χ^2^ test for independence to assess differences in the prevalence of attempted breastfeeding before hospital discharge between women delivering in Matamoros and women delivering in Cameron County. Statistical significance was set at α = .05. We could not assess differences between either community and the overall combined sample because the combined data were a composite of the data from the individual communities, and observations were not independent.

To quantify the difference in the odds of attempted breastfeeding before hospital discharge by selected sociodemographic characteristics, health behaviors, and perinatal/delivery experiences, we used logistic regression analysis. Variables that were statistically significant (*P* <.05) in the bivariate analyses were included in the multivariate logistic regression models. In addition to these variables, we included variables that approached significance (*P* <.10 and >.05) in the logistic regression models to account for other potential sources of variance and confounders. Only variables with at least 30 cases unweighted per level were considered in the logistic regression analysis. Models were estimated separately for Cameron County and Matamoros and for the combined sample.

## Results

Distribution of demographic and behavioral variables, by place of residence, is presented in Table 2. Although only 5.0% of all Matamoros residents delivered in the United States, all Cameron County residents, with the exception of 1, delivered their infants in the United States. Nearly all residents in Matamoros completed the interview in Spanish. In Cameron County, the language in which the interview was completed was approximately evenly distributed between English and Spanish. The ethnicity of respondents from Cameron County was primarily Hispanic. Despite having a greater percentage of respondents report early entry into prenatal care, the percentage of low-birth-weight infants was higher in Cameron County than in Matamoros.

### Sociodemographic characteristics and attempted breastfeeding

In Matamoros, the rate of attempted breastfeeding before hospital discharge was higher among respondents with fewer years of education (Table 3). In the multivariate model, respondents in Matamoros with fewer than 8 years of education were nearly twice as likely to attempt breastfeeding as were respondents with more than 12 years of education (Table 4). Respondents in Matamoros with 8 to 12 years of education were almost 3 times as likely to attempt breastfeeding as were respondents with more than 12 years of education.

The prevalence of attempted breastfeeding was 81.9% among the women who lived in Matamoros, compared with 63.7% among the women who lived in Cameron County (data not shown). In the multivariate analysis, residing in Matamoros was associated with nearly a 2-fold increase in the odds of attempted breastfeeding before hospital discharge (Table 4).

In Cameron County, non-Hispanic women had a greater prevalence of attempted breastfeeding before hospital discharge than did Hispanic women. After adjusting for other factors, Hispanic women who resided in Cameron County had decreased odds of attempted breastfeeding before hospital discharge compared with non-Hispanic women in Cameron County.

### Perinatal experiences

Women who delivered vaginally had increased prevalence and odds of attempted breastfeeding in Matamoros and in the 2 communities combined (Tables 3 and 4). Prevalence of attempted breastfeeding was higher among women who delivered vaginally in Cameron County, but odds of attempted breastfeeding among these same women were not significant in the multivariate model.

In Matamoros, Cameron County, and the 2 communities combined, women who delivered a low-birth-weight infant (ie, <2,500 g) had a lower rate of attempted breastfeeding before hospital discharge than did women who delivered an infant of normal weight (ie, ≥2,500 g) (Table 3). In each of the multivariate logistic regression models, women who delivered a low-birth-weight infant were approximately 75% less likely to have attempted breastfeeding before hospital discharge than were women who delivered infants of normal weight (Table 4).

Women with any previous live births had a higher prevalence of attempting to breastfeed in Matamoros, but the same was not true in Cameron County (Table 3). In the multivariate logistic regression model, women in Matamoros who had a previous live birth were nearly twice as likely to attempt breastfeeding before hospital discharge as were women who had not had a previous live birth. However, this was not true of the women in Cameron County (Table 4).

In Cameron County and in the 2 communities combined, women who indicated that their pregnancy was intended had a higher prevalence of attempted breastfeeding before hospital discharge than did women who indicated that their pregnancy was unintended (Table 3). In the multivariate logistic regression model, the odds of attempted breastfeeding before hospital discharge were 50% lower among women who identified their pregnancy as unintended than among women with intended pregnancies. In the combined model, the decrease in odds was 34% (Table 4).

## Discussion

The prevalence of attempted breastfeeding before hospital discharge was nearly 20 percentage points higher in Matamoros than in Cameron County, and the adjusted odds of attempted breastfeeding before hospital discharge among Matamoros residents were nearly twice the odds among Cameron County residents. The rates we report in Matamoros and Cameron County are lower than previously reported state rates on both sides of the US-Mexico border. Rates of ever breastfeeding in Texas have been reported to be 75% (27), and rates of ever breastfeeding in northern Mexico, an area that includes Tamaulipas, have been reported to be 91% (28). These statistics suggest that breastfeeding rates in border areas may be lower than statewide rates.

Most women's first breastfeeding experience occurs in the hospital environment, and exclusive breastfeeding rates decline substantially after hospital discharge. Failure to establish breastfeeding during the hospital stay is a factor in breastfeeding cessation following hospital discharge (29). Although the importance of breastfeeding is highlighted in the United States at the national level in documents like *Healthy People 2010* (30), no national policy exists to support breastfeeding.

Unlike the United States, Mexico has federal regulations that support breastfeeding. Mexico's official norms for breastfeeding, or Ministry of Health clinical practice guidelines (*La Norma Oficial Mexicana*), support breastfeeding initiation within the first 2 hours after delivery when conditions permit; support and facilitate breastfeeding on infant demand; and include standards, criteria, and procedures that promote and protect exclusive breastfeeding (eg, standards for training and personnel, requirements that medical units provide appropriate conditions to enable mothers to practice exclusive breastfeeding and to educate mothers about the benefits of exclusive breastfeeding) (31). CAALMA Curso, a training program for hospital personnel, has been implemented throughout Mexico to support these federal regulations (32).

A study in Texas among WIC (Supplemental Nutrition Program for Women, Infants, and Children) recipients who breastfed found that 55% of women were not informed that breastfeeding could occur at the demand of the infant, 56% of women did not initiate breastfeeding in the first hour after delivery, and 74% of women reported their infants were fed something in addition to breast milk (33). At least 1 US study found that hospitals that adopted supportive breastfeeding policies have more patients who breastfeed and who breastfeed longer (34).

Another difference between the United States and Mexico is the provision of infant formula in hospitals. Federal regulations in Mexico restrict the distribution of formula in the hospital, the free distribution or promotion of breast milk substitutes by medical units, and the distribution of incentives to health care providers from the manufacturers of breast milk substitutes (31). Furthermore, in September 2007, an agreement (*Acuerdo con Productores de Alimentos y Fórmulas Infantiles*) was reached with Mexican health officials and manufacturers of infant formula to restrict the distribution of formula in the hospital, the free distribution or promotion of breast milk substitutes by medical units, and the distribution of incentives to health care providers from the manufacturers of breast milk substitutes (Cuitlahuac Ruiz Matus, MD, written communication, February 14, 2008). Similar regulations are not in place in the United States. In Texas, nearly three-quarters of women who received WIC benefits reported receiving formula before hospital discharge.

Women in our study who delivered infants weighing ≥2,500 g had increased odds of breastfeeding in Matamoros, Cameron County, and in the combined sample. Women who delivered vaginally had increased odds of breastfeeding in Matamoros and in the combined sample. These findings are consistent with previous studies and identify opportunities for cross-border collaboration (35-38). Cross-border work groups may design continuing education for providers or develop protocols and best practices that address the unique breastfeeding needs of women who deliver infants weighing <2,500 g or who have a cesarean delivery. In Mexico, strategies and best practices to increase breastfeeding among women in these vulnerable groups could be disseminated through the CAALMA Curso.

Women who did not intend to become pregnant had decreased odds of attempted breastfeeding before hospital discharge in Cameron County and in the combined sample. Previous research on this association has shown mixed results (39-41). At least 1 study found an association between pregnancy intention and attempted breastfeeding before hospital discharge in the United States (40). However, studies in Bolivia and Paraguay did not find an association between these variables (40,41). Although practitioners should discuss breastfeeding with all pregnant women, more education and counseling may be needed for women whose pregnancy is unintended.

Our study has 2 limitations. The first limitation was with regard to duration of hospital stay. Women who delivered in Matamoros were typically discharged on the same day of their delivery, and women who delivered in Cameron County typically remained hospitalized for up to 48 hours after delivery. Therefore, women who delivered in Cameron County had more time to attempt to breastfeed than did women who delivered in Matamoros. Despite this difference, women who delivered in Matamoros still attempted to breastfeed at a greater rate than did women who delivered in Cameron County, suggesting that differences on the basis of place of residence would be larger if the length of hospital stay after delivery were equal. The second limitation is that previous infant feeding method was not included in the questionnaire. However, the exclusion of this variable most likely affected point estimates within the 2 locations rather than the estimate associated with place of residence.

If breastfeeding rates reached the goal of 75% in the early postpartum period established in *Healthy People 2010* (29), the potential cost savings could exceed $1 billion (10). Results from this study are encouraging for Matamoros but indicate a need for additional breastfeeding support and messages in Cameron County. Because of the cross-border mobility of the population, joint US-Mexico strategies to promote breastfeeding are needed.

## Figures and Tables

**Table 1:** Study Measures, Brownsville-Matamoros Sister City Project, Brownsville, Texas, and Matamoros, Tamaulipas, Mexico, August 21-November 9, 2005

**Indicator**	**Question**	**Definition**
**Sociodemographic characteristics**		
Ethnicity	Do you consider yourself to be of Hispanic or Latino origin or descent?	Respondents who resided in Mexico were classified as *Hispanic*. Respondents who resided in the United States were classified on the basis of their responses to the question (ie, *Hispanic* or *Non-Hispanic*). Therefore, ethnicity could not be included in analyses limited to Matamoros only.
Age	What is your birth date?	Age was calculated using date of birth and date of interview. On the basis of mean age (25 y), women were divided into 2 groups (*<25* *y* and *≥25 y*).
Marital status	What is your marital status now?	Marital status was dichotomized: 1) married or living as married and 2) not married. Women who indicated that they were "married" or "live-in significant other/consensual union" were classified as *married*. Women who indicated they were "single," "widowed," or "divorced/separated" were classified as *not married*.
Education	What was the *highest* grade or level of school you have *completed* or the *highest degree* you have *received*?	Education was divided into 3 groups based on the number of years in school each woman had completed (*<8 y*, *8-12 y*, *>12 y*).
Employment status	During the *3 months before* you got pregnant with your new baby, which of the following best describes your employment status?	Women who responded "employed for wages, money, or a paycheck" or "self-employed" were categorized as *employed*. Women who responded "out of work" were categorized as *unemployed*. Women who responded "homemaker," "student," "retired," or "unable to work" were categorized as *not in labor force*.
Mother's place of birth	In what country were you born?	Possible responses included "Mexico," "the United States," "other," and "don't know." Women who responded "other" and "don't know" were combined into a single group.
Place of residence	In what country do you live?	Possible responses included "United States," "Mexico," "both," "don't know/not sure," and "refused." Women who responded "both" or "don't know/not sure" and women whose place of residence was not recorded were assigned a place of residence on the basis of their place of delivery.
Place of delivery	NA	Place of delivery was recorded at the time of interview. Interviews were conducted in hospitals in Cameron County, Texas, or Matamoros, Tamaulipas, Mexico.
Language spoken during interview	The language in which the interview was conducted was recorded by the interviewer.	The language spoken during the interview was used as a proxy for the primary language of the participant. A respondent who used any Spanish during the interview was classified as *Spanish speaker*. A respondent who did not use any Spanish during the interview was classified as *English speaker*.
**Health behaviors**		
Smoking status	Have you smoked at least 100 cigarettes in the past 2 years? A pack has 20 cigarettes. In the *3 months* *before* you got pregnant, how many cigarettes did you smoke on an average day? A pack has 20 cigarettes.	Tobacco use was created from 2 variables that measured *smoked 100 cigarettes in the past 2 years* and *number of cigarettes smoked on an average day 3 months before this pregnancy*. A respondent who had not smoked 100 cigarettes in the past 2 years or had smoked zero cigarettes on an average day 3 months before this pregnancy was classified as a *nonsmoker*. A respondent who had smoked 100 cigarettes in the past 2 years or who had smoked any cigarettes on an average day 3 months before this pregnancy was classified as a *smoker*.
Alcohol use	Have you had any alcoholic drinks in the past 2 years? A drink is 1 glass of wine, wine cooler, can or bottle of beer, shot of liquor, or mixed drink. During the *3 months before* you got pregnant, how many alcoholic drinks did you have in an average week?	Alcohol use was created from 2 variables that measured any alcohol in the last 2 years and frequency of alcohol use 3 months before this pregnancy. A respondent who drank any alcohol in the past 2 years and who drank any alcohol during the 3 months before this pregnancy was classified as an alcohol *user*. A respondent who had not had any alcohol in the last 2 years and who drank in the last 2 years but did not drink alcohol during the 3 months before this pregnancy was classified as an alcohol *nonuser*.
**Perinatal/delivery experiences**		
Infant birth weight	Birth weight was recorded from the birth certificate.	Infants weighing <2,500 g were coded as *low-birth-weight*. Infants weighing ≥2,500 g were coded as *normal-weight*.
Parity	Including your most recent pregnancy, how many times have you been pregnant?	Responses were dichotomized: 1) women who had *no previous live births* and 2) women who had *any* (ie, 1 or more) *previous live births*.
Health care coverage during pregnancy	During this pregnancy did you have any kind of health care coverage plan or insurance plan?	Women who responded yes were coded as *has coverage,* and women who responded no were coded as *does not have coverage.*
Delivery method	Please tell me which one of the following statements best describes how your new baby was delivered. Vaginally; You went into labor, but had to have a C-section; You didn't go into labor and had to have a C-section.	Possible responses included "vaginal delivery," "cesarean section with labor," and "cesarean section without labor." Responses of "cesarean section with labor" and "cesarean section without labor" were combined.
Prenatal care	How many weeks or months pregnant were you when you had your first visit for prenatal care? Don't count a visit that was only for a pregnancy test or only for Special Supplemental Nutrition Program for Women, Infants, and Children (known in the United States as WIC).	Responses were standardized to weeks. Women who had their first prenatal visit during their first trimester were classified as having received *early* prenatal care. Women who did not receive prenatal care and those that had their first visit during their second or third trimesters were classified as having received late or no prenatal care (*late or none*).
Pregnancy intention	Thinking back to *just before* you got pregnant with your new baby, how did you feel about becoming pregnant?	Responses were dichotomized into groups representing *intended pregnancy* ("you wanted to be pregnant sooner," "you wanted to be pregnant then") and *unintended pregnancy* ("you wanted to be pregnant later," "you didn't want to be pregnant then or at any time in the future").

Abbrevations: NA, not applicable.

**Table 2:** Distribution of Demographic and Behavioral Variables, Brownsville-Matamoros Sister City Project, Brownsville, Texas, and Matamoros, Tamaulipas, Mexico, August 21-November 9, 2005^a^

Characteristic	Total Sample		Matamoros		Cameron County		*P* Value^b^

	Unweighted No. of Respondents (n = 946)	Weighted No. of Respondents (%) (n = 5,094)	Unweighted No. of Respondents (n = 489)	Weighted No. of Respondents (%) (n = 2,764)	Unweighted No. of Respondents (n = 457)	Weighted No. of Respondents (%) (n = 2,331)	
**Place of delivery**							
United States	483	2,464 (48.4)	27	139 (5.0)	456	2,325 (99.7)	<.001
Mexico	463	2,630 (51.6)	462	2,624 (95.0)	1	6 (0.3)	
**Ethnicity^c^**							
Hispanic	883	4,772 (95.0)	489	2,765 (100.0)	394	2,009 (88.9)	<.001
Non-Hispanic	49	250 (5.0)	NA	NA	49	250 (11.1)	
**Age, y**							
<25	456	2,464 (48.4)	248	1,403 (50.8)	208	1,062 (45.6)	.10
≥25	490	2,630 (51.6)	241	1,361 (49.2)	249	1,269 (54.4)	
**Marital status**							
Not married	165	864 (17.1)	46	258 (9.4)	119	607 (26.3)	<.001
Married	773	4,187 (82.9)	440	2,489 (90.6)	333	1,699 (73.7)	
**Education, y**							
<8	211	1,163 (23.0)	156	883 (32.0)	55	280 (12.1)	<.001
8-12	416	2,262 (44.7)	248	1,404 (50.9)	168	858 (37.2)	
>12	313	1,638 (32.3)	84	471 (17.1)	229	1,168 (50.7)	
**Employment status**							
Employed	454	2,450 (48.5)	238	1,350 (48.9)	216	1,100 (47.9)	.01
Unemployed	72	380 (7.5)	24	134 (4.9)	48	246 (10.7)	
Not in labor force	412	2,223 (44.0)	226	1,274 (46.2)	186	949 (41.4)	
**Mother's place of birth**							
United States	253	1,291 (25.5)	2	11 (0.4)	195	995 (43.2)	<.001
Mexico	678	3,724 (73.6)	483	2,729 (99.0)	251	1,280 (55.5)	
Other/don't know	9	47 (0.9)	3	17 (0.6)	6	30 (1.3)	
**Primary language**							
English	239	1,217 (23.9)	3	17 (0.6)	236	1,200 (51.5)	<.001
Spanish	707	3,877 (76.1)	486	2,746 (99.4)	221	1,131 (48.5)	
**Smoking status**							
Smoker	60	319 (6.3)	24	135 (4.9)	36	184 (7.9)	.01
Nonsmoker	883	4,759 (93.7)	464	2,622 (95.1)	419	2,137 (92.1)	
**Alcohol use**							
User	223	1,172 (23.1)	66	372 (13.5)	157	801 (65.5)	<.001
Nonuser	720	3,906 (76.9)	422	2,386 (86.5)	298	1,520 (34.5)	
**Health care coverage during pregnancy**							
Has coverage	653	3,525 (69.3)	337	1,915 (69.4)	316	1,610 (69.2)	.93
Does not have coverage	291	1,558 (30.7)	151	843 (30.6)	140	715 (30.8)	
**Delivery method**							
Cesarean	415	2,234 (43.9)	217	1,224 (44.4)	198	1,010 (43.3)	.68
Vaginal	530	2,855 (56.1)	271	1,534 (55.6)	259	1,320 (56.7)	
**Infant birth weight**							
Low (<2,500 g)	64	341 (6.7)	24	136 (5.0)	40	205 (8.8)	.01
Normal (≥2,500 g)	878	4,731 (93.3)	462	2,610 (95.0)	416	2,121 (91.2)	
**Parity**							
Any previous live births	638	3,430 (67.3)	317	1,793 (64.9)	321	1,637 (70.3)	.06
No previous live births	308	1,664 (32.7)	172	971 (35.1)	136	693 (29.7)	
**Prenatal care**							
Early	496	2,647 (52.8)	217	1,224 (45.0)	279	1,423 (62.0)	<.001
Late or none	435	2,366 (47.2)	264	1,494 (55.0)	171	872 (38.0)	
**Pregnancy intention**							
Intended to get pregnant	451	2,444 (48.4)	259	1,466 (53.3)	192	978 (42.5)	<.001
Did not intend to get pregnant	487	2,608 (51.6)	228	1,286 (46.7)	259	1,322 (57.5)	

Abbreviation: NA, not applicable.

a Columns do not all total to number in sample size because of missing data.

b χ^2^ test used to determine statistical differences.

c All respondents who resided in Mexico were classified as Hispanic. Therefore, ethnicity could not be included in analyses limited to Matamoros only.

**Table 3:** Prevalence of Attempted Breastfeeding Before Hospital Discharge, Brownsville-Matamoros Sister City Project, Brownsville, Texas, and Matamoros, Tamaulipas, Mexico, August 21-November 9, 2005

Characteristic	Matamoros		Cameron County		Total Sample	

	Weighted % (95% CI)	*P* Value	Weighted % (95% CI)	*P* Value	Weighted % (95% CI)	*P* Value
**Place of delivery **						
United States	77.8 (62.2-93.4)	.59	63.6 (58.9-68.4)	.27	64.4 (60.0-68.9)	<.001
Mexico	82.1 (79.1-82.3)		100.0		82.1 (80.0-84.3)	
**Ethnicity^a^**						
Hispanic	81.9 (79.7-84.1)	NA	61.7 (56.0-67.4)	.007	73.4 (70.8-75.9)	.28
Non-Hispanic	NA		79.7 (68.7-90.7)		79.7 (68.7-90.7)	
**Age, y**						
<25	83.9 (80.3-87.4)	.15	62.6 (54.8-70.5)	.65	74.7 (70.6-78.8)	.41
≥25	79.8 (76.4-83.2)		64.7 (59.6-69.7)		72.5 (69.7-75.3)	
**Marital status**						
Not married	84.7 (76.4-92.8)	.51	58.9 (50.3-67.4)	.14	66.6 (60.2-73.0)	.01
Married	81.9 (79.7-84.1)		65.2 (60.4-70.0)		75.1 (72.9-77.4)	
**Education, y**						
<8	85.4 (80.4-90.3)	<.001	65.3 (54.6-76.0)	.94	80.5 (76.0-85.1)	<.001
8-12	85.5 (82.0-89.0)		63.8 (55.7-71.9)		77.3 (73.6-80.9)	
>12	65.4 (56.2-74.7)		63.0 (56.8-69.2)		63.7 (58.7-68.7)	
**Employment status**						
Employed	79.0 (75.7-82.3)	.06	61.2 (54.5-67.9)	.31	71.0 (67.7-74.4)	.10
Unemployed	91.9 (82.2- 100.0)		58.5 (44.1-72.9)		70.3 (59.7-80.9)	
Not in labor force	84.2 (79.8-88.6)		67.7 (60.5-74.9)		77.2 (73.1-81.3)	
**Mother's place of birth**						
United States	50.0 (0.0-100.0)	.06	61.1 (54.9-67.3)	.45	61.0 (54.8-67.2)	.001
Mexico	82.1 (79.7-84.4)		66.6 (60.3-73.0)		77.9 (75.6-80.2)	
Other/don't know	100.0		67.0 (32.6-100.0)		78.8 (55.5-100.0)	
**Primary language**						
English	0	.06	60.7 (55.7-65.7)	.09	59.8 (54.9-64.8)	<.001
Spanish	82.4 (80.2-84.6)		67.0 (60.4-73.5)		77.9 (75.6-80.1)	
**Smoking status**						
Smoker	70.6 (54.0-87.1)	.17	61.0 (45.7-76.4)	.70	65.1 (54.0-76.2)	.12
Nonsmoker	82.6 (80.4-84.8)		64.0 (59.4-68.7)		74.3 (72.0-76.6)	
**Alcohol use**						
User	78.9 (71.8-86.0)	.35	62.5 (56.2-68.8)	.60	67.7 (62.7-72.7)	.005
Nonuser	82.5 (80.2-84.9)		64.5 (59.0-70.0)		75.5 (73.2-77.8)	
**Health care coverage during pregnancy**						
Has coverage	81.1 (78.5-83.6)	.39	63.4 (57.9-68.9)	.81	73.0 (70.2-75.8)	.47
Does not have coverage	83.6 (78.7-88.5)		64.3 (57.9-70.6)		74.7 (70.9-78.5)	
**Delivery method**						
Cesarean	71.4 (68.1-74.6)	<.001	59.6 (54.0-65.3)	.03	66.1 (62.2-70.0)	<.001
Vaginal	90.5 (85.1-95.9)		66.9 (61.0-72.8)		79.6 (76.4-82.7)	
**Infant birth weight**						
Low (<2,500 g)	54.2 (34.5-73.8)	.01	30.1 (15.3-44.9)	.001	39.7 (27.9-51.6)	<.001
Normal (≥2,500 g)	83.2 (81.0-85.4)		67.1 (62.8-71.5)		76.0 (73.9-78.1)	
**Parity**						
Any previous live births	86.2 (83.2-89.3)	.002	60.5 (55.3-65.7)	.01	74.0 (71.3-76.7)	.68
No previous live births	73.8 (67.7-80.0)		71.4 (63.4-79.3)		72.8 (68.0-77.5)	
**Prenatal care**						
Early	78.5 (74.1-82.9)	.08	65.7 (60.7-70.6)	.09	71.6 (68.3-74.9)	.07
Late or none	84.4 (80.0-87.9)		60.9 (54.5-67.4)		75.8 (72.6-78.9)	
**Pregnancy intention**						
Intended to get pregnant	80.7 (77.4-84.0)	.27	72.0 (67.1-76.8)	.003	77.2 (74.5-79.8)	.009
Did not intend to get pregnant	83.5 (80.0-87.0)		57.6 (50.1-65.1)		70.4 (66.4-74.3)	

Abbreviations: CI, confidence interval; NA, not applicable.

a In Matamoros, all women were considered to be of Hispanic ethnicity. Therefore, no data are reported for non-Hispanic ethnicity, and the χ^2^ is not calculated.

**Table 4:** Odds of Attempted Breastfeeding Before Hospital Discharge, Brownsville-Matamoros Sister City Project, Brownsville, Texas, and Matamoros, Tamaulipas, Mexico, August 21-November 9, 2005

Characteristic	Matamoros Model^a^ **AOR** (95% CI)	Cameron County Model^a^AOR (95% CI)	Matamoros and Cameron County Model^a^AOR (95% CI)
**Place of residence**			
United States	—	—	Ref
Mexico	—	—	1.93 (1.31-2.84)
**Ethnicity**			
Hispanic	—	0.42 (0.21-0.85)	—
Non-Hispanic	—	Ref	—
**Marital status**			
Not married	—	—	1.10 (0.79-1.55)
Married	—	—	Ref
**Education level, y**			
<8	1.94 (1.11-3.42)	—	1.27 (0.79-2.06)
8-12	2.91 (1.59-5.32)	—	1.39 (0.94-2.05)
>12	Ref	—	Ref
**Employment status**			
Employed	Ref	—	Ref
Unemployed	1.70 (0.42-6.89)	—	1.03 (0.56-1.91)
Not in labor force	1.01 (0.57-1.77)	—	1.29 (0.92-1.82)
**Language**			
English	—	Ref	Ref
Spanish	—	1.40 (0.99-1.99)	1.35 (0.92,2.00)
**Alcohol use**			
User	—	—	0.99 (0.73-1.36)
Nonuser	—	—	Ref
**Delivery method**			
Cesarean	Ref	Ref	Ref
Vaginal	3.63 (1.91-6.90)	1.15 (0.83-1.60)	1.98 (1.43-2.75)
**Infant birth weight**			
Low (<2,500 g)	0.21 (0.11-0.44)	0.22 (0.10-0.50)	0.26 (0.15-0.44)
Normal (≥2,500 g)	Ref	Ref	Ref
**Parity**			
Any previous live births	1.96 (1.18-3.25)	0.64 (0.40-1.03)	—
No previous live births	Ref	Ref	—
**Prenatal care**			
Early	0.89 (0.49-1.63)	1.13 (0.83-1.52)	0.97 (0.74-1.29)
Late or none	Ref	Ref	Ref
**Pregnancy intention**			
Intended to get pregnant	—	Ref	Ref
Did not intend to get pregnant	—	0.50 (0.33-0.77)	0.66 (0.49-0.89)

Abbreviations: AOR, adjusted odds ratio; CI, confidence interval.

a Each column represents a separate logistic regression model and all variables included in the model. Variables that satisfy eligibility criteria for inclusion in one model may not for another model. Dashes indicate that a variable did not meet the criteria for inclusion in that model but did for 1 or more of the other models.

## References

[1] Anderson JB (2003). The U.S.-Mexico border: a half century of change. Soc Sci J.

[2] Peach J, Williams J (2003). Population dynamics of the US-Mexican border region.

[3] Brandon JE, Crespin F, Levy C, Reyna DM, Bruhn JG, Brandon JE (1997). Border health issues. Border health: challenges along the U.S.-Mexico border.

[4] (1988). Health care: availability in the Texas-Mexico border area. GAO Document # HRD-89-12.

[5] Gartner LM, Morton J, Lawrence RA, Naylor AJ, O'Hare D, Schanler RJ (2005). Breastfeeding and the use of human milk. Pediatrics.

[6] Goldman AS, Hopkinson JM, Rassin DK (2007). Benefits and risks of breastfeeding. Adv Pediatr.

[7] Ball TM, Wright AL (1999). Health care costs of formula-feeding in the first year of life. Pediatrics.

[8] Montgomery DL, Splett PL (1997). Economic benefit of breast-feeding infants enrolled in WIC. J Am Diet Assoc.

[9] Tuttle CR, Dewey KG (1996). Potential cost savings for Medi-Cal, AFDC, food stamps, and WIC programs associated with increasing breast-feeding among low-income Hmong women in California. J Am Diet Assoc.

[10] Weimer D (2001). The economic benefits of breastfeeding: a review and analysis. Food Assistance and Nutrition Research Report No. 13.

[11] Harley K, Stamm NL, Eskenazi B (2007). The effect of time in the U.S. on the duration of breastfeeding in women of Mexican descent. Matern Child Health J.

[12] Gibson MV, Diaz VA, Mainous AG, Geesey ME (2005). Prevalence of breastfeeding and acculturation in Hispanics: results from NHANES 1999-2000 study. Birth.

[13] Libbus MK (2000). Breastfeeding attitudes in a sample of Spanish-speaking Hispanic American women. J Hum Lact.

[14] Kaiser LL, Melgar-Quiñonez HR, Lamp CL, Johns MC, Harwood JO (2001). Acculturation of Mexican-American mothers influences child feeding strategies. J Am Diet Assoc.

[15] Byrd TL, Balcazar H, Hummer RA (2001). Acculturation and breast-feeding intention and practice in Hispanic women on the US-Mexico border. Ethn Dis.

[16] Rassin DK, Markides KS, Baranowski T, Richardson CJ, Mikrut WD, Bee DE (1994). Acculturation and the initiation of breastfeeding. J Clin Epidemiol.

[17] Rassin DK, Markides KS, Baranowski T, Bee DE, Richardson CJ, Mikrut WD (1993). Acculturation and breastfeeding on the United States-Mexico border. Am J Med Sci.

[18] Merewood A, Brooks D, Bauchner H, MacAuley L, Mehta SD (2006). Maternal birthplace and breastfeeding initiation among term and preterm infants: a statewide assessment for Massachusetts. Pediatrics.

[19] Singh GK, Kogan MD, Dee DL (2007). Nativity/immigrant status, race/ethnicity, and socioeconomic determinants of breastfeeding initiation and duration in the United States, 2003. Pediatrics.

[20] Gibson-Davis CM, Brooks-Gunn J (2006). Couples' immigration status and ethnicity as determinants of breastfeeding. Am J Public Health.

[21] Celi AC, Rich-Edwards JW, Richardson MK, Kleinman KP, Gillman MW (2005). Immigration, race/ethnicity, and social and economic factors as predictors of breastfeeding initiation. Arch Pediatr Adolesc Med.

[22] Balcazar H, Trier CM, Cobas JA (1995). What predicts breastfeeding intention in Mexican-American and non-Hispanic white women? Evidence from a national survey. Birth.

[23] Smith JC, Mhango CG, Warren CW, Rochat RW, Huffman SL (1982). Trends in the incidence of breastfeeding for Hispanics of Mexican origin and Anglos on the U.S.-Mexico border. Am J Public Health.

[24] Barriers to binational cooperation in public health between Texas and Mexico.

[25] (2000). Mortality profiles of the sister communities on the United States — Mexico border, 2000 edition.

[26] McDonald JA, Johnson CH, Smith R, Folger SG, Chavez AL, Mishra N (2008). Reproductive health surveillance in the US-Mexico border region, 2003-2006: the Brownsville-Matamoros Sister City Project for Women's Health. Prev Chronic Dis.

[27] (2007). Centers for Disease Control and Prevention. Breastfeeding trends and updated national health objectives for exclusive breastfeeding — United States, birth years 2000-2004. MMWR Morb Mortal Wkly Rep.

[28] González-Cossío T, Moreno-Macías H, Rivera JA, Villalpando S, Shamah-Levy T, Monterrubio EA (2003). Breast-feeding practices in Mexico: results from the Second National Nutrition Survey 1999. Salud Publica Mex.

[29] Harrod-Wild K (2007). Lessons from the latest infant feeding survey. J Fam Health Care.

[30] (2000). Healthy People 2010. 2nd ed. With understanding and improving health and objectives for improving health. 2 vols.

[31] Atención de la mujer durante el embarazo, parto y puerperio y del recién nacido. Criterios y procedimientos para la prestación del servicio. Norma Oficial Mexicana. NOM-007-SSA2-1993.

[32] Argomedo AL, Bribiesca F, Reyes-Vazquez H (2005). CAALMA Curso, avanzado de apoyo a la lactancia materna. Conepeme.

[33] (2006). Breastfeeding beliefs, attitudes, and practices in the Texas WIC population: findings from the 2006 Infant Feeding Survey.

[34] Murray EK, Ricketts S, Dellaport J (2007). Hospital practices that increase breastfeeding duration: results from a population-based study. Birth.

[35] Meier PP, Brown LP (1996). Breastfeeding for mothers and low-birth-weight infants. Nurs Clin North Am.

[36] Callen J, Pinelli J (2005). A review of the literature examining the benefits and challenges, incidence and duration, and barriers to breastfeeding in preterm infants. Adv Neonatal Care.

[37] Rowe-Murray HJ, Fisher JR (2002). Baby friendly hospital practices: cesarean section is a persistent barrier to early initiation of breastfeeding. Birth.

[38] Rowe-Murray HJ, Fisher JR (2001). Operative intervention in delivery is associated with compromised early mother-infant interaction. BJOG.

[39] Taylor JS, Cabral HJ (2002). Are women with an unintended pregnancy less likely to breastfeed?. J Fam Pract.

[40] Shapiro-Mendoza C, Selwyn BJ, Smith DP, Sanderson M (2005). Parental pregnancy intention and early childhood stunting: findings from Bolivia. Int J Epidemiol.

[41] Shapiro-Mendoza CK, Selwyn BJ, Smith DP, Sanderson M (2007). The impact of pregnancy intention on breastfeeding duration in Bolivia and Paraguay. Stud Fam Plann.

